# Relative Humidity on Mars: New Results From the Phoenix TECP Sensor

**DOI:** 10.1029/2019JE006080

**Published:** 2019-11-06

**Authors:** E. Fischer, G. M. Martínez, N. O. Rennó, L. K. Tamppari, A. P. Zent

**Affiliations:** ^1^ Department of Climate and Space Sciences and Engineering University of Michigan Ann Arbor MI USA; ^2^ Lunar and Planetary Institute Universities Space Research Association Houston TX USA; ^3^ Jet Propulsion Laboratory California Institute of Technology Pasadena CA USA; ^4^ NASA Ames Research Center Mountain View CA USA

**Keywords:** Mars, Phoenix, TECP, relative humidity, water vapor, water cycle

## Abstract

In situ measurements of relative humidity (RH) on Mars have only been performed by the Phoenix (PHX) and Mars Science Laboratory (MSL) missions. Here we present results of our recalibration of the PHX thermal and electrical conductivity probe (TECP) RH sensor. This recalibration was conducted using a TECP engineering model subjected to the full range of environmental conditions at the PHX landing site in the Michigan Mars Environmental Chamber. The experiments focused on the warmest and driest conditions (daytime) because they were not covered in the original calibration (Zent et al., 2010, https://doi.org/10.1029/2009JE003420) and previous recalibration (Zent et al., 2016, https://doi.org/10.1002/2015JE004933). In nighttime conditions, our results are in excellent agreement with the previous 2016 recalibration, while in daytime conditions, our results show larger water vapor pressure values. We obtain vapor pressure values in the range ~0.005–1.4 Pa, while Zent et al. (2016, https://doi.org/10.1002/2015JE004933) obtain values in the range ~0.004–0.4 Pa. Our higher daytime values are in better agreement with independent estimates from the ground by the PHX Surface Stereo Imager instrument and from orbit by Compact Reconnaissance Imaging Spectrometer for Mars. Our results imply larger day‐to‐night ratios of water vapor pressure at PHX compared to MSL, suggesting a stronger atmosphere‐regolith interchange in the Martian arctic than at lower latitudes. Further, they indicate that brine formation at the PHX landing site via deliquescence can be achieved only temporarily between midnight and 6 a.m. on a few sols. The results from our recalibration are important because they shed light on the near‐surface humidity environment on Mars.

## Key Points

- We have recalibrated the relative humidity sensor of the Mars Phoenix lander
- We obtain water vapor pressure values in the range ~0.005–1.4 Pa, while in previous recalibrations, values in the range ~0.004–0.4 Pa
- Our results show a two‐order‐of‐magnitude diurnal variation of water vapor pressure, suggesting a strong atmosphere‐regolith interchange

## 1 Introduction

The Phoenix (PHX) mission arrived at Mars' north polar region (68.2°N, 234.2°E) in 2008 to study the history of water and search for habitable environments (Smith et al., 2008). It operated for 151 sols (*L*_*s*_ = 78°–148°), exceeding the mission primary requirement of 90 sols. Among a wide range of instruments analyzing the polar environment such as a meteorological station (Taylor et al., 2008), a “telltale” wind sensor (Holstein‐Rathlou et al., 2010), and a LIDAR (Whiteway et al., 2008), PHX carried the thermal and electrical conductivity probe (TECP) to support the search for liquid water on Mars (Zent et al., 2009).

The TECP is one of the instruments of the microscopy, electrochemistry, and conductivity analyzer payload (Hecht et al., 2008) on the PHX lander. It is mounted on the robotic arm (RA) of the lander and was designed to study the regolith's thermal properties and water content by performing six different types of measurements: air temperature, atmospheric relative humidity (RH), and the regolith's temperature, thermal conductivity, volumetric heat capacity, electrical conductivity, and dielectric permittivity (Zent et al., 2009). The TECP consists of a single electronics box, fitted with four needles which can be inserted into the Martian regolith for conducting measurements. The RH sensor is mounted on the outside of the TECP's structure.

The original calibration of the TECP atmospheric RH sensor was performed at the University of Washington Mars Atmospheric Simulation Chamber (Zent et al., 2009), using a pair of frost point hygrometers (a Buck CR‐1 chilled‐mirror hygrometer and an EdgeTech DewPrime I chilled‐mirror hygrometer) as a reference. More than 50,000 measurements were conducted, covering frost points ranging from 194 to 263 K and temperatures ranging from 208 to 303 K (with corresponding RH values in range of ~0% to ~55%). Then, a calibration function of the form *RH = f (DNRH, T*_*b*_ *)* was produced, where *RH* is the processed RH, *DNRH* is the raw RH output of the sensor, and *T*_*b*_ is the temperature of the TECP electronics board where the RH sensor is mounted (Zent et al., 2009). Unless otherwise noted, we refer to RH with respect to water ice when using RH in this manuscript. We refer the reader to Rivera‐Valentín et al. (2018) for a clarification between RH values obtained with respect to liquid and with respect to ice as well as for the set of equations used in both cases.

The values of *DNRH* and *T*_*b*_ covered in the original calibration only partially overlap the environmental conditions later found at the PHX landing site (Zent et al., 2016). Specifically, neither was the RH sensor calibrated at *T*_*b*_ < 208 K, nor was it calibrated at high *T*_*b*_ and low *DNRH* values observed at midday on Mars. Therefore, processed *RH* values obtained from measurements around noon (when *T*_*b*_ is high and *DNRH* is low), and at dawn (when *T*_*b*_ is the lowest) presented large uncertainties, and were removed from the NASA Planetary Data System (PDS) in 2010.

The calibration function was revised twice to correct for inaccuracies at the lowest temperatures (Zent et al., 2012, 2016). In order to improve the original at *T*_*b*_ < 208 K, flight data from known conditions in sols 86, 91, 103, 104, and 122 taken between 00:00 and 04:00 were added to the calibration data obtained in the laboratory. On each of these sols, PHX LIDAR measurements indicated that the Martian atmosphere was saturated throughout the lowest ~1 km after 23:00 (Whiteway et al., 2009), and the humidity and temperature of the saturated air (*RH* = 100%) were used to estimate the frost point and augment the original calibration data set. In addition, and since the original flight instrument calibration was performed against hygrometers that measured frost point temperatures (*T*_*f*_) rather than *RH*, the revised calibration function was revised to take the form *T*_*f*_ *= f (DNRH, T*_*b*_ *)* (Zent et al., 2016). The processed humidity values from this latest recalibration were posted back into NASA's PDS in 2016.

Here we further improve the TECP RH sensor's calibration by significantly augmenting the preflight (laboratory data) and flight‐data calibration data sets. We use our Michigan Mars Environmental Chamber (MMEC) to recalibrate the TECP. An engineering model of the TECP subjected to the entire range of atmospheric pressure, temperature, and preprocessed *DNRH* values measured by the PHX lander is used to conduct the recalibration. We focus on the warmest and driest conditions (daytime) because data at these conditions were neither covered in the preflight nor in the revised calibration.

Our laboratory apparatus and calibration methodology are described in section 2. The results of our recalibration are shown in section 3, while a comparison with previous calibration efforts and other independent measurements is shown in section 4. A discussion of our results is presented in section 5. A summary of the conclusions is presented in section 6.

## 2 Methodology

### 2.1 The MMEC

**Figure 1:**
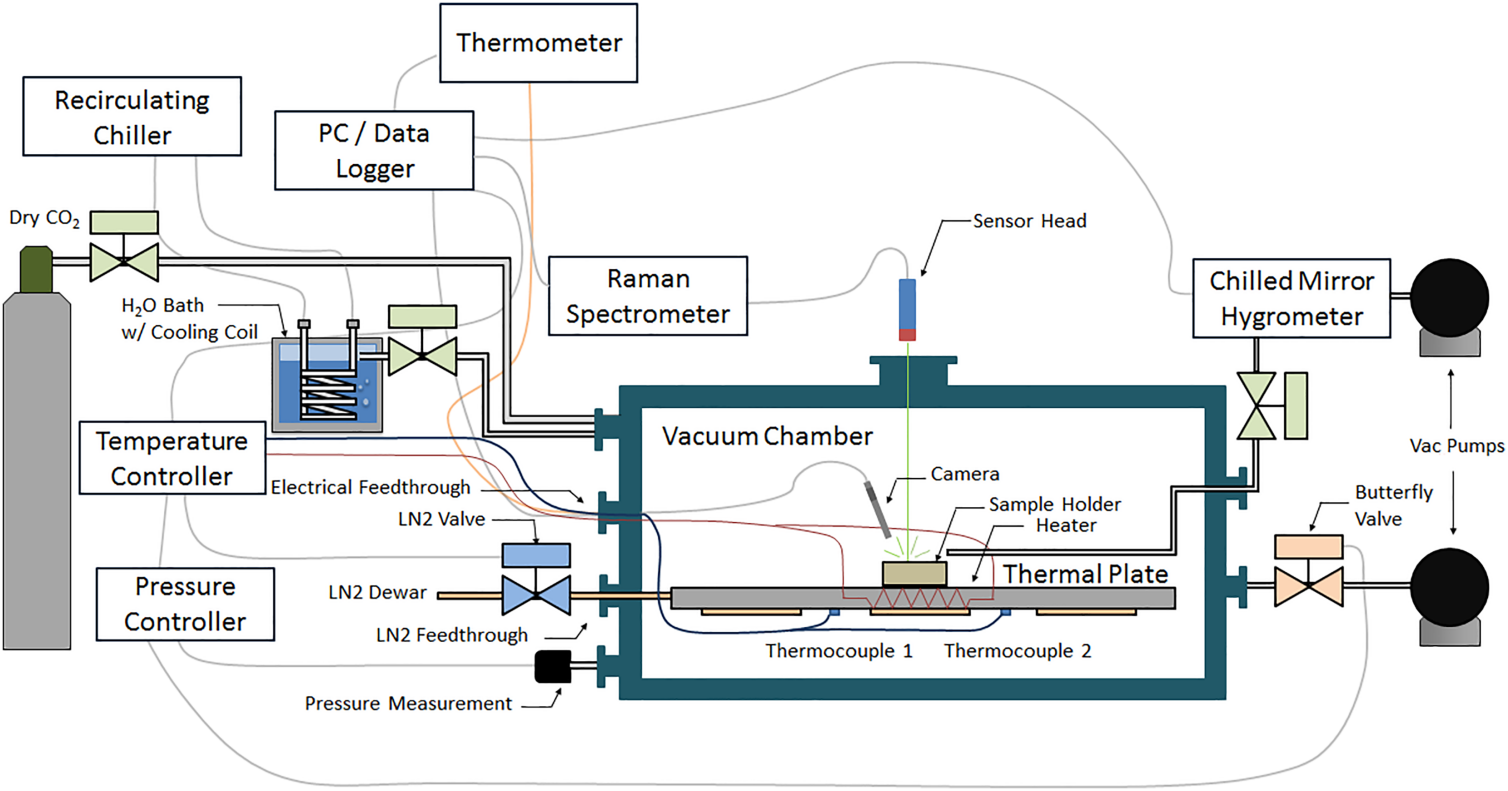
Sketch of the Michigan Mars Environmental Chamber (MMEC). It can simulate the entire range of atmospheric pressure, temperature, and relative humidity encountered at the Phoenix landing site. The MMEC has been used to augment the calibration of the relative humidity sensors onboard the Mars Science Laboratory, Mars 2020, and ExoMars 2020 missions (Hieta et al., 2019).

Our recalibration of the TECP RH sensor was performed in the MMEC, a cylindrical chamber with internal diameter of 64 cm and length of 160 cm (Figure 1). Because of its unique capabilities, the MMEC has been used to augment the calibration of the RH sensors of the Mars Science Laboratory (MSL), Mars 2020, and ExoMars 2020 missions (Hieta et al., 2019). The MMEC can simulate the entire range of environmental conditions encountered at the PHX landing site, including pressure between 720 and 860 Pa, temperature from 180 to 270 K, and RH from ~0% to >100% (Taylor et al., 2010; Davy et al., 2010; Tamppari et al., 2010; Whiteway et al., 2009). This allows us to recalibrate the sensor within the entire range of in situ conditions it experienced on Mars.

The MMEC has an automated feedback control system that uses a thermal plate with embedded cartridge heaters and a liquid nitrogen cooling loop to control the temperature. Water vapor is added to the chamber through a temperature and pressure‐controlled H_2_O bath. The RH of the MMEC atmosphere can be adjusted to selected values by controlling the flow from the water bath into it. The local RH is sampled right at the location of the TECP RH sensor and measured by an independent frost point hygrometer (a Buck CR‐1A chilled‐mirror hygrometer, similar to the one used in the preflight calibration). Finally, the pressure is controlled by an automated feedback control system.

### 2.2 The New TECP RH Calibration Function

**Figure 2:**
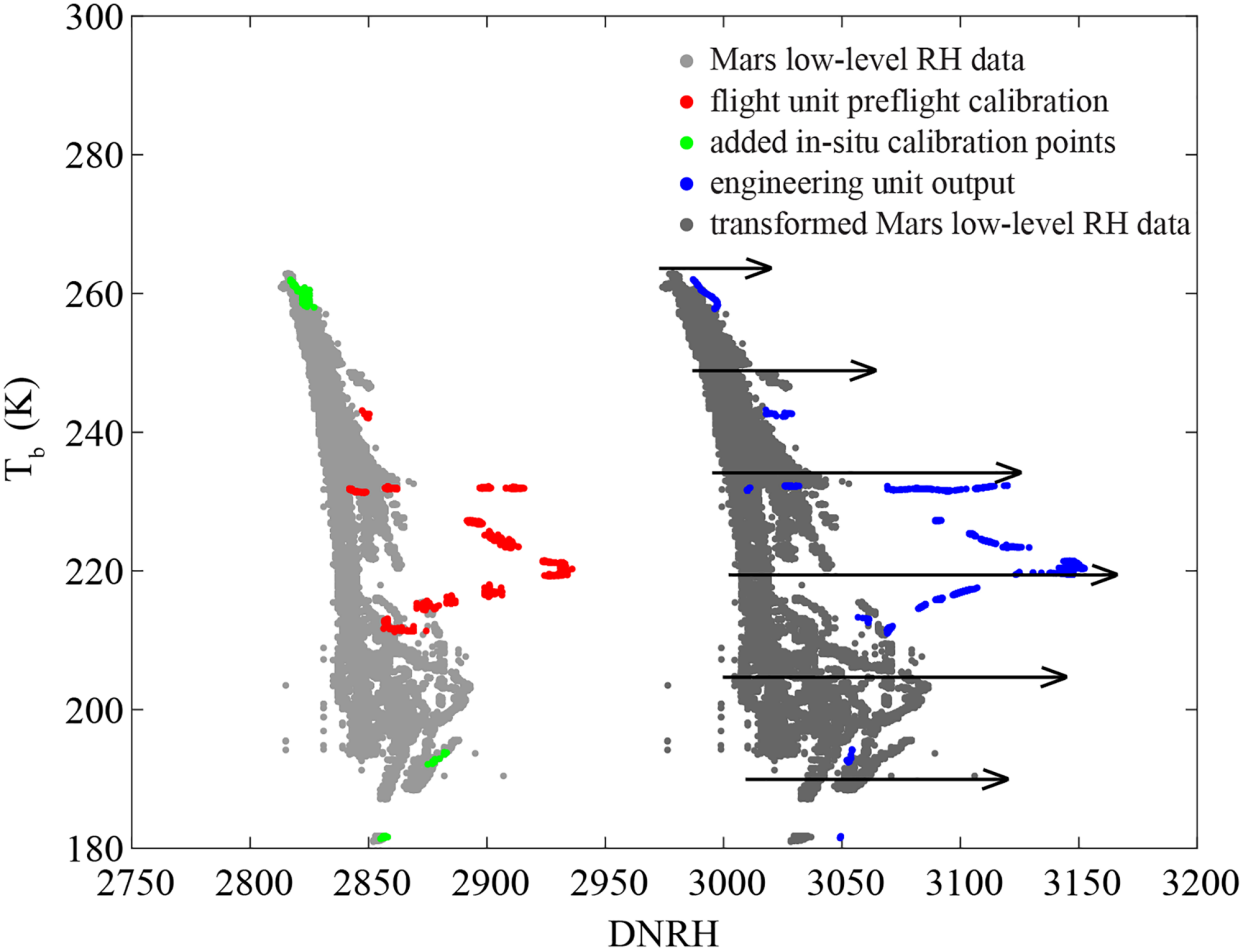
The thermal and electrical conductivity probe (TECP) preflight calibration data (red) only partially overlaps the recorded relative humidity (RH) measurements at the Phoenix landing site (light gray). We use the output of a TECP engineering unit (blue) that matches the environmental conditions of the preflight calibration (red) in terms of *T*_*b*_ and *T*_*f*_ and additional known landing site conditions (green) to transform the in situ measurements range (light gray) into the range of the engineering unit (dark gray). We then use the entire output of the engineering unit (symbolized by the arrows) to cover the entire range of *T* and *RH* conditions (dark gray) to calibrate the engineering unit and find a recalibration for the flight unit.

The TECP engineering unit that we use in our experiments is a spare of the instrument flown on PHX. To characterize its dynamic range, we simulated the entire range of PHX landing site temperatures and RH values, with RH ranging from near 0% to saturated conditions, recording the raw RH output (*DNRH*) of the TECP engineering unit. The dynamic range of *DNRH* values differs between both units at the same exact board temperature (*T*_*b*_) and frost point temperature (*T*_*f*_; and therefore of *RH*). This difference is within manufacturer specifications but has to be accounted for in our recalibration. This is illustrated in Figure 2, where the red points represent the initial preflight calibration in terms of measured *T*_*b*_ and *DNRH* and the blue points represent the *DNRH* output of the engineering unit at the same conditions of *T*_*b*_ and *T*_*f*_ as in the red points.

To account for the difference in dynamic range of both TECP units for recalibration purposes, we obtain a “translation function” *g* of the form as follows:

$${\text{DNRH}}_{\mathrm{eu}}=g\left({\text{DNRH}}_{\mathrm{fu}},T_{b}\right),$$

which relates the raw engineering unit *RH* output *DNRH*_*eu*_ (Figure 2, blue) to that of the flight unit *DNRH*_*fu*_ (red) at the same environmental conditions of pressure, *T*_*b*_ and *T*_*f*_. To improve the accuracy of this translation function, we use values of in situ measurements of *T*_*b*_ and *DNRH* as additional calibration points (Figure 2, green). Here, there are two distinct groups of those points. One group is at the lowest observed *T*_*b*_ range during the second half of the mission when near‐surface fog was observed (Whiteway et al., 2009). This allows us to safely assume saturated atmospheric RH conditions (*RH* = 100%), and therefore, we can use measured atmospheric temperatures as actual *T*_*f*_ values (Zent et al., 2016). The other group is at the highest observed in situ *T*_*b*_ values during midday. Neither were these values covered by the original (preflight) calibration (Zent et al., 2009) nor by the revised calibration (Zent et al., 2016). In this group of points, we do not have independent in situ measurements that can provide the actual *T*_*f*_ values at the highest temperatures. Therefore, in this case, we impose upper bounds for *T*_*f*_ ranging from ~213 to 225 K corresponding to expected upper bound in atmospheric water vapor partial pressure (*e*) values ranging from ~1 to ~5 Pa. This corresponds to *RH* < ~2.3% at the highest measured temperatures (*T*_*b*_ ~ 260 K; top green points in Figure 2). The rationale for selecting such an upper‐bound range is given below.

These *T*_*f*_ values represent conservative upper bounds. Satellite and surface‐based retrievals of precipitable water vapor column abundance (PWC) at the PHX landing site indicate near‐surface, daytime *e* values well below 5 Pa (Tamppari et al., 2010). This is further supported by results from numerical modeling (Savijärvi & Määttänen, 2010). Specifically, daytime retrievals of PWC from the PHX Surface Stereo Imager (SSI) show maximum values of around 50 pr‐μm (corresponding to *e* ~ 2.5 Pa in a well‐mixed daytime atmosphere; Figure 3 in Tamppari et al., 2010). Similar upper bounds at the PHX location at daytime were measured from orbit by the thermal emission spectrometer at equivalent water vapor pressure values of up to 1.0 Pa, the Compact Reconnaissance Imaging Spectrometer for Mars (CRISM), and the Observatoire pour la Minéralogie, l'Eau, les Glaces, et l'Activité, while the Mars Atmospheric Water Detector (MAWD) measured historic maximum values of ~80 pr‐μm (corresponding to *e* ~ 4 Pa), later corrected to values comparable to thermal emission spectrometer, CRISM, and Observatoire pour la Minéralogie, l'Eau, les Glaces, et l'Activité following the use of an updated spectroscopic database and improved atmospheric model assumptions (Fedorova et al., 2010; Pankine et al., 2009; Pankine & Tamppari, 2015).

After consideration of historic satellite retrievals at the PHX site, we initially impose a very conservative upper bound of ~5 Pa (*T*_*f*_ ~ 225 K; corresponding to ~100 pr‐μm). To test the impact of our *T*_*f*_ assumption and further refine this upper bound, we have performed sensitivity studies of the results of the calibration function with respect to the upper bound values for *T*_*f*_ we selected. Analyses of these sensitivity studies indicate that *T*_*f*_ values between ~216 and 220 K (~1.5 and 2.5 Pa) result in the most accurate calibration function. A value of *T*_*f*_ ~ 218 K (~2 Pa) was selected as the upper bound in the determination of the calibration function. We discuss this in more detail in section 3.3.

Once the two sets of calibration points are added, we obtain the following translation function with a coefficient of determination of 86.2%:

(1) $${\text{DNRH}}_{\mathrm{eu}}=-997.8+1.411{\text{DNRH}}_{\mathrm{fu}}+1.097\times 10^{-2}T_{b}.$$

This low‐order function represents the difference of output between the engineering and flight units of the TECP, without the unrealistic variations in the preflight calibration values that may occur when using higher‐order polynomials for interpolation. We then apply this translation function to the raw data obtained at the PHX landing site with the flight unit (Figure 2, light gray), resulting in the dark gray cloud in Figure 2. This would be the output of the engineering unit of the TECP RH sensor, if it had conducted measurements concurrently with the flight sensor at the PHX landing site.

As the final step of the recalibration of the TECP RH sensor, we cover the range of *T*_*b*_ and *DNRH* shown in dark gray in Figure 2. To achieve this, we place the TECP engineering unit inside our environmental chamber in good thermal contact with the chamber's thermal plate. We then lower the pressure inside the environmental chamber to 850 Pa of CO_2_. Next, we dry out the chamber and sensor, before lowering the sensor's temperature to 181 K, the lowest temperature encountered by the TECP RH sensor throughout the PHX mission (see Figure 2, black). While keeping the temperature constant, we start adding water vapor to the chamber's environment, increasing the RH from ~0% to 100%. We repeat this process for the entire temperature range while measuring the raw output of the TECP humidity sensor and the frost point independently with a chilled mirror hygrometer. This new calibration data set covers >250,000 data points at *T*_*b*_ values between 180 and 263 K ranging from ~0% to 100% RH at each temperature step. Potential errors with respect to the experimental data are discussed in section 3.3.

We use the experimentally obtained data to obtain a new calibration function *f* of the following form based on previous studies of TECP calibration functions (Zent et al., 2012, 2016), with a coefficient of determination of 95.1%, showing that this function fits the calibration data well:

(2) $$T_{f,\mathrm{hyg}}=f\left({\text{DNRH}}_{\mathrm{eu}},T_{b}\right)=a_{1}{\text{DNRH}}_{\mathrm{eu}}^{2}+a_{2}{\text{DNRH}}_{\mathrm{eu}}+a_{3}\frac{{\text{DNRH}}_{\mathrm{eu}}}{T_{b}}+a_{4}T_{b}^{2}+a_{5}T_{b}+a_{6}\;a_{1}=-5.346\times 10^{-4}K\;a_{2}=4.090\;K\;a_{3}=-146.4\;K^{2}\;a_{4}=4.531\times 10^{-2}\;K^{-1}\;a_{5}=-28.82\;a_{6}=-1122\;K.$$

We can then apply the translation and calibration function to the in situ *DNRH* values measured by the TECP to obtain the recalibrated frost point values at the PHX landing site:

$$T_{f}=f\left(g\left({\text{DNRH}}_{\mathrm{fu}},T_{b}\right)\right).$$

Equivalently to the frost‐point temperatures obtained, we can calculate the water vapor pressure using the saturation vapor pressure with respect to ice (Buck, 1981) as follows:

(3) $$e=e_{s,i}\left(T_{f}\right)=611.35\;\exp\frac{22.542\left(T_{f}-273.16\right)}{T_{f}+0.32}.$$

More recent equations for the saturation vapor pressure with respect to ice result in values very similar to those using the equation by Buck (1981). For example, using the equation by Wagner et al. (2011) results in a maximum difference in *e* of 0.8%.

## 3 Results

### 3.1 Temporal Coverage of the TECP RH Sensor

A comprehensive list of observations made by the TECP including type (air vs. ground), elevation, intent, and target location is shown in Table 1 of Zent et al. (2010). Here we give a summary of the temporal coverage of the TECP RH sensor to provide context for the results of the calibration shown in section 3.2.

**Figure 3:**
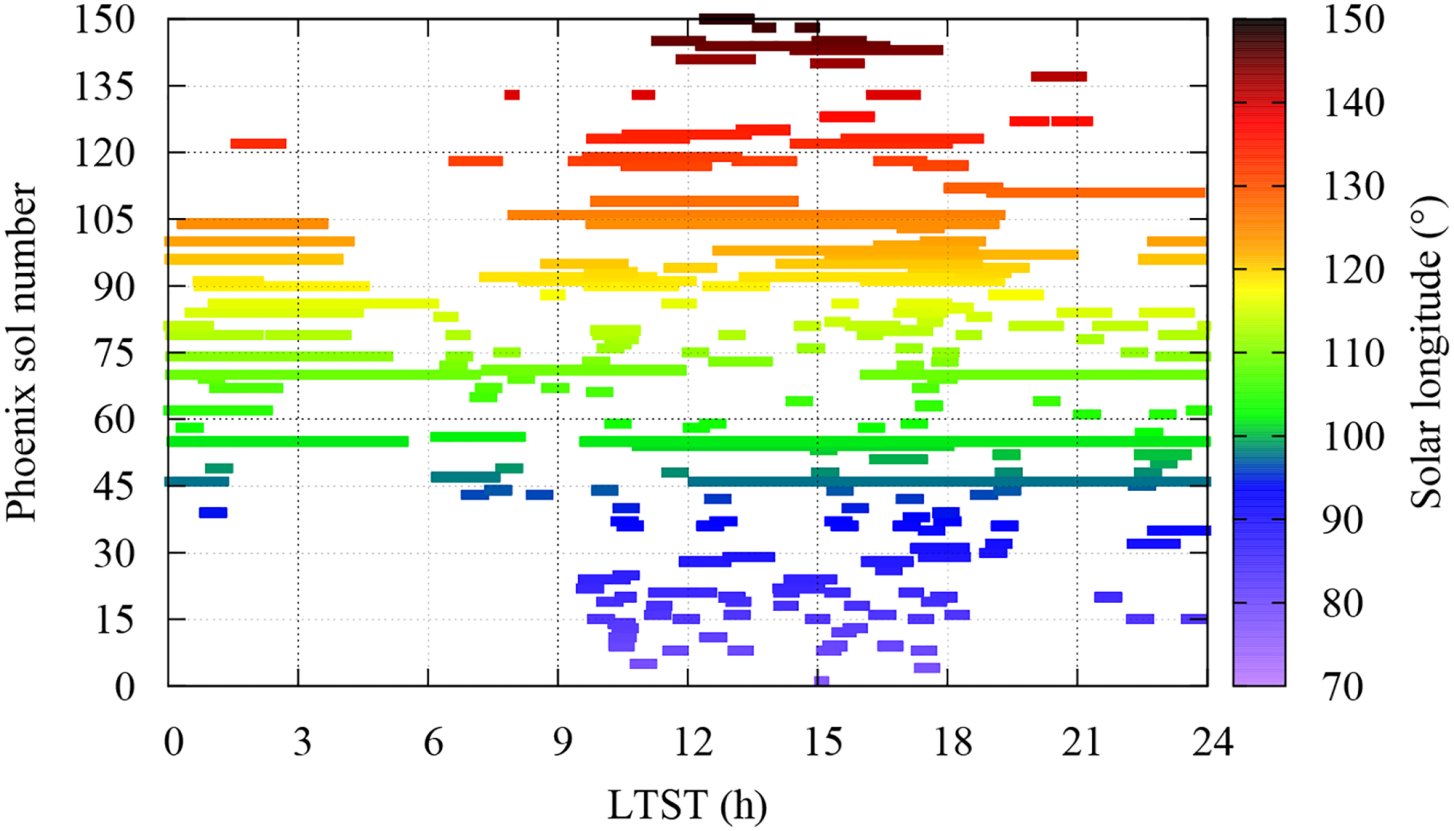
Temporal coverage of the thermal and electrical conductivity probe relative humidity (RH) sensor as a function of local true solar time and sol number, with solar longitude color‐coded. In‐soil measurements were taken on sols 46–47, 54–55, 69–71, 86, 98, 103–104, 111, 119, 122–124, and 149–150. On the remaining sols, atmospheric RH measurements were conducted at heights ranging from 0 to ~2.2 m.

The TECP RH sensor operated for nearly the entire duration of the PHX mission, from sol 1 (*L*_*s*_ ~ 77°) to 150 (*L*_*s*_ ~ 148°), but not continuously. This was due to competitive demands on the RA, where the TECP was mounted. Typically, measurements were taken with a sampling rate of 1.2 s during ~30′‐long blocks a few times per sol (Figure 3). Additionally, extended blocks with durations ranging from ~30′ to ~20 hr were frequently taken. This was particularly the case for in‐soil measurements, which were taken on sols 46–47, 54–55, 69–71, 86, 98, 103–104, 111, 119, 122–124, and 149–150 as part of specific campaigns aimed at studying the electrical properties of the regolith (Zent et al., 2010). On the remaining sols, measurements were taken in the air at heights ranging from 0 to ~2.2 m, depending on the position of the RA.

The TECP RH measurement strategy resulted in a fairly complete diurnal coverage when the entire mission is considered. The most densely covered period was 10 a.m.–6 p.m. (Figure 3), with an average of ~30 hr of measurements per hourly bin. In contrast, the 4–6 a.m. period was the least densely covered, with an average of ~8 hr of measurements per hourly bin. However, day‐to‐day variations in the environmental conditions were strong, particularly during the second half of the mission when the atmospheric pressure and temperature were rapidly declining as the polar night approached (Davy et al., 2010; Taylor et al., 2010). During that period, the solar insolation dropped abruptly, resulting in deposition of atmospheric CO_2_ and H_2_O on the ground (Martínez et al., 2017). Therefore, assessments of the humidity environment at the PHX landing site, particularly on diurnal timescales, must take into consideration the limited temporal coverage and the strong day‐to‐day variations in the environmental conditions.

### 3.2 New Values of Water Vapor Pressure, Frost Point, and RH

**Figure 4:**
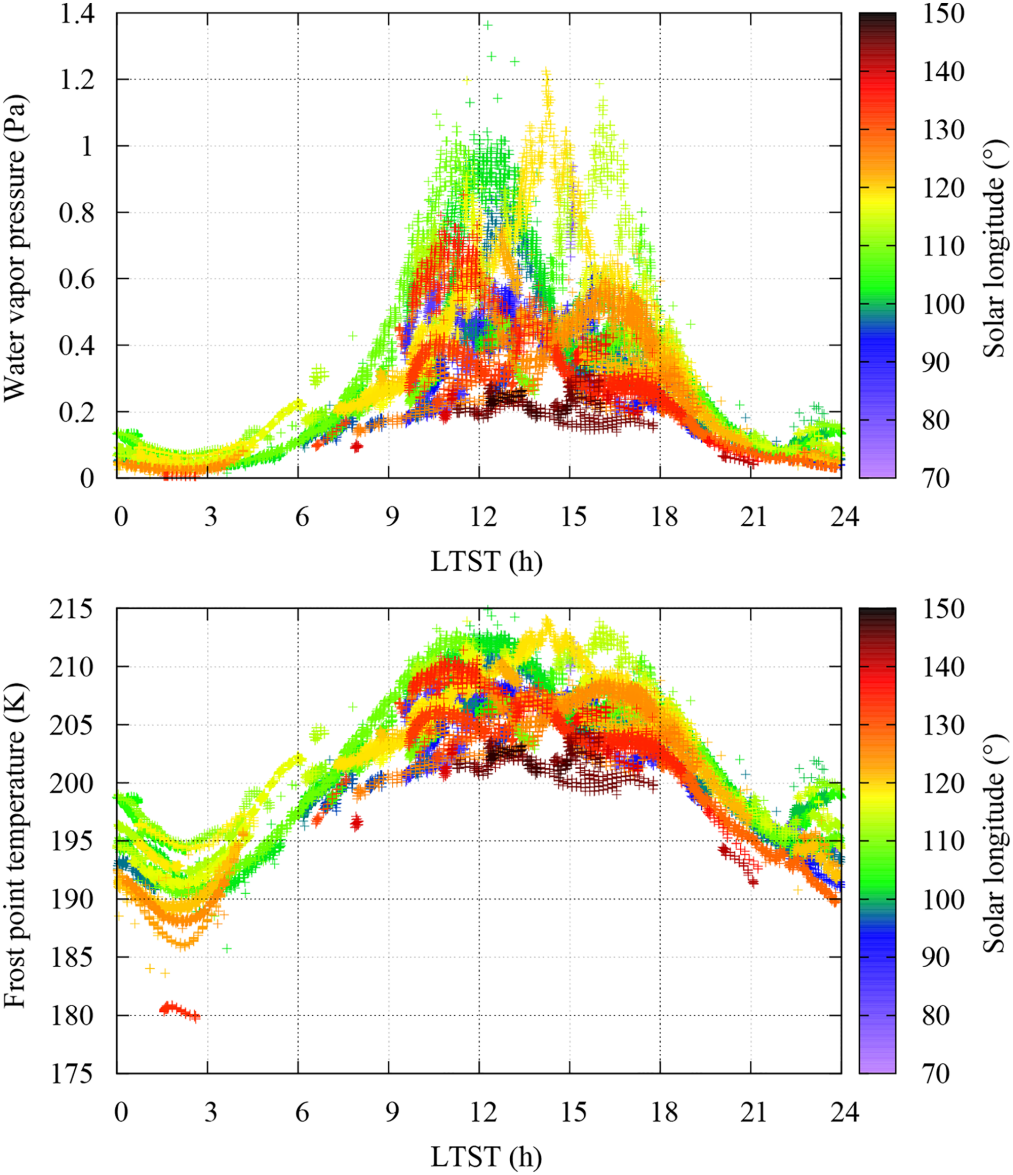
The recalibrated thermal and electrical conductivity probe relative humidity sensor measurements at the Phoenix landing site color‐coded by *L*_*s*_ as water vapor pressure (top) and frost point temperature (bottom) over local time.

Figure 4 shows the water vapor pressure (top) and frost‐point (bottom) values obtained based on our recalibration as a function of local true solar time (LTST), with *L*_*s*_ shown using color code. Values of *e* range between ~0.005 (*T*_*f*_ ~180 K) and 1.4 Pa (*T*_*f*_ ~ 215 K), which were measured, respectively, on sol 122 (*L*_*s*_ ~ 133°) at ~2 a.m. and on sol 54 (*L*_*s*_ ~ 101°) around noon (Figure 3). Although the TECP did not operate continuously, a nearly complete diurnal coverage was achieved on sol 55 (Figure 3). On this sol, the water vapor pressure underwent a diurnal variation of two orders of magnitude, from around ~0.01 Pa at 3 a.m. to ~1 Pa at noon. While the TECP needles were inserted into the ground on this sol, the humidity sensor was located a few centimeters above the ground due to the geometry of the TECP unit.

The highest maximum diurnal values of water vapor pressure occur between sols 60 (*L*_*s*_ ~ 104°) and 90 (*L*_*s*_ ~ 118°; green and yellow colors in Figure 4), in excellent agreement with contemporaneous satellite retrievals of water vapor column abundance (Tamppari et al., 2010). Maximum diurnal values decrease in the late mission after sol 110 (*L*_*s*_ ~ 128°), when the temperatures dropped and the water vapor was deposited on the surface (brown colors in Figure 4; Whiteway et al., 2009; Davy et al., 2010).

**Figure 5:**
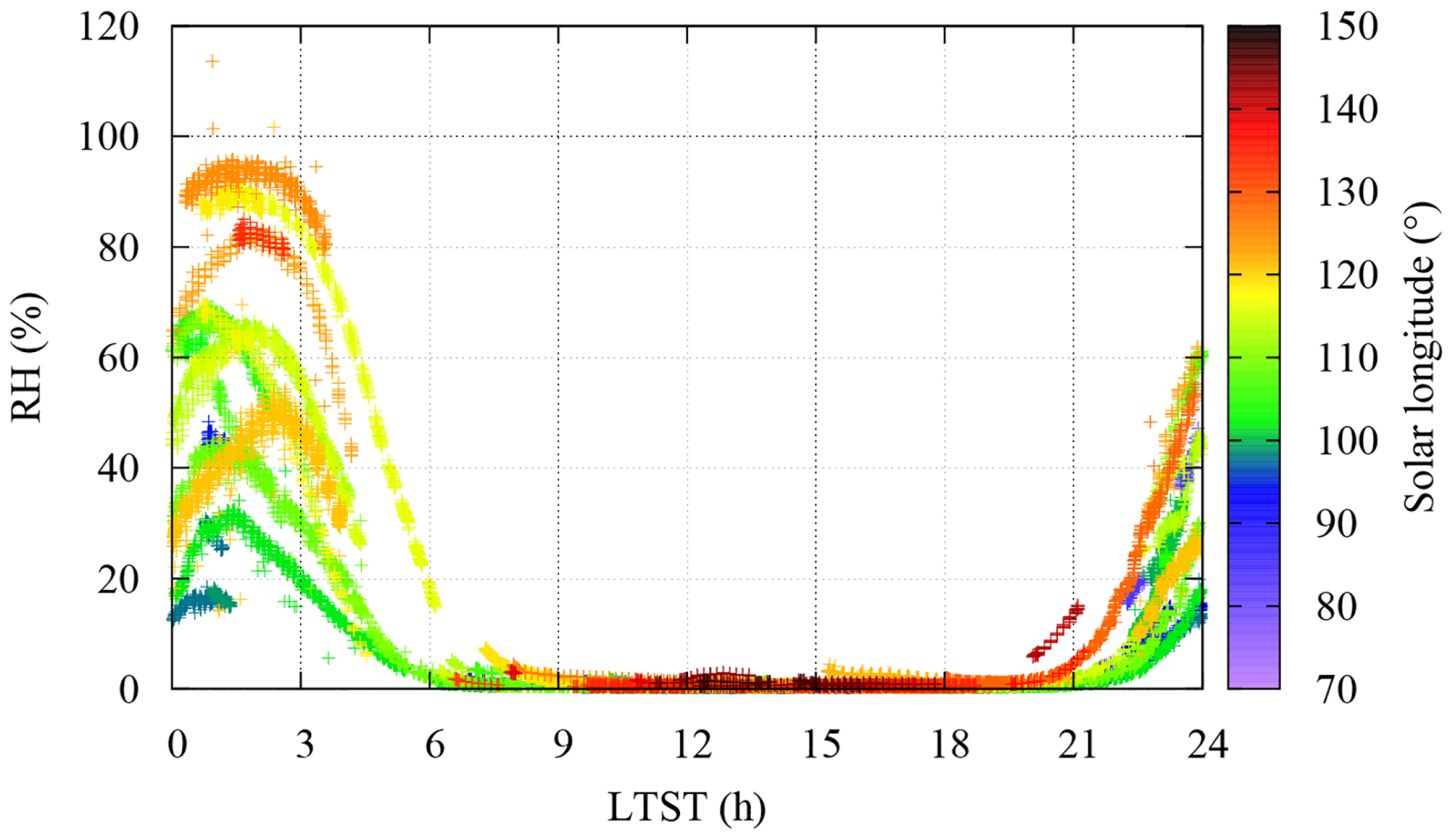
The recalibrated thermal and electrical conductivity probe relative humidity based on frost‐point and board temperature measurements at the Phoenix landing site color‐coded by *L*_*s*_ as local relative humidity at the sensor location.

Figure 5 shows the diurnal cycle of *RH* throughout the entire mission, color‐coded by *L*_*s*_. The RH shown here is obtained using the board temperature of the RH sensor as the local reference temperature and shows values close to saturation levels between sols 90 (*L*_*s*_ ~ 118°) and 100 (*L*_*s*_ ~ 123°). The RH can be obtained at different heights using independent temperature measurements, assuming a constant value of water vapor pressure in the vertical profile of the near‐surface atmosphere. For instance, Meteorological Station (MET) temperatures at 2 m above the ground (Davy et al., 2010), which are typically colder than concurrent *T*_*b*_ values due to heating of the TECP electronics, result in *RH* values that surpass saturation levels between sols ~70 (*L*_*s*_ ~ 108°) and 110 (*L*_*s*_ ~ 128°). This is in excellent agreement with independent observations by the RA camera and the LIDAR, showing nighttime frost formation from about sol 70, and fall streaks and fog reaching all the way to the ground from sol 109, respectively (Smith et al., 2009; Whiteway et al., 2009).

### 3.3 Error Analysis

The error in water vapor pressure can be estimated based on random instrument errors during the calibration experiments, as well as on the implications in the assumption of the upper bound value of water vapor pressure at the highest observed temperatures, necessary for determining the translation function. Using equations (1) and (2), we obtain the random error in the frost point temperatures as follows:

(4) $$\delta T_{f}=\sqrt{{\left(\frac{\partial T_{f}}{\partial \mathrm{DNR}H_{\mathrm{eu}}}\text{δDNR}H_{\mathrm{eu}}\right)}^{2}+{\left(\frac{\partial T_{f}}{\partial T_{b}}\delta T_{b}\right)}^{2}}$$

with

(5) $$\text{δDNR}H_{\mathrm{eu}}=\sqrt{{\left(\frac{\partial \mathrm{DNR}H_{\mathrm{eu}}}{\partial \mathrm{DNR}H_{\mathrm{fu}}}\text{δDNR}H_{\mathrm{fu}}\right)}^{2}+{\left(\frac{\partial \mathrm{DNR}H_{\mathrm{eu}}}{\partial T_{b}}\delta T_{b}\right)}^{2}}.$$

The random board temperature measurement error at the TECP RH sensor has a maximum value of 0.75 K throughout the entire range of environmental conditions simulated, while the *DNRH* output of the flight instrument varies by one unit under constant environmental conditions. Using the resulting error in frost‐point temperature and equation (3), we find that the error in water vapor pressure ranges from 4% to 16% of the actual water vapor pressure values.

**Figure 6:**
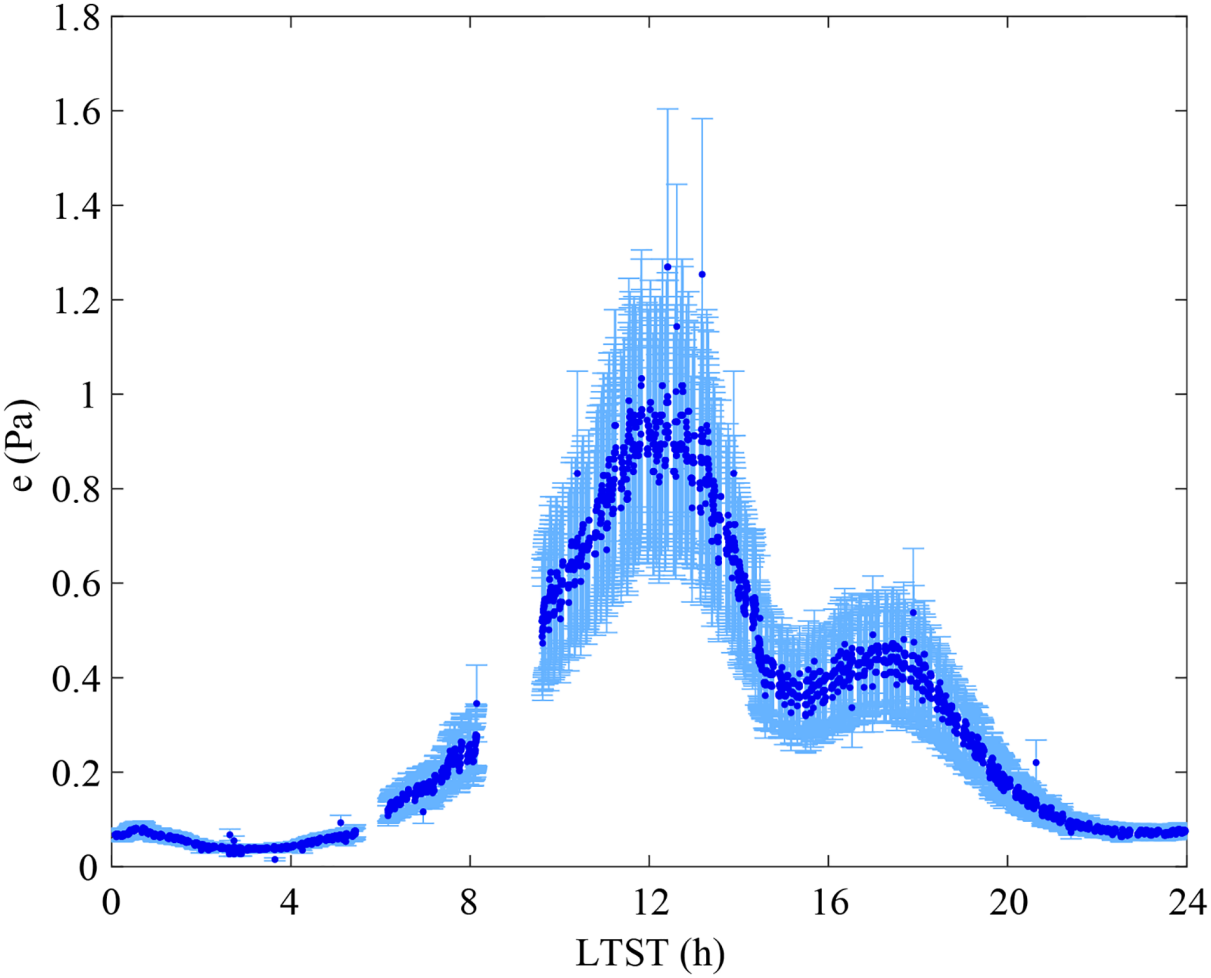
Recalibrated Phoenix thermal and electrical conductivity probe measurements on sols 55 and 56 with error bars based on instrument errors and errors due to the upper‐bound assumption for water vapor pressure at the highest temperatures.

The other main source of inaccuracy in our recalibration results is the value of the assumed upper bound for the water vapor pressure at the PHX landing site, at the warmest temperatures. Varying the upper bound used for our calibration function between 1 and 5 Pa results in slight variations in the maximum water vapor pressure values obtained using this new calibration function. To further refine the upper bound, we disregard values below 1.5 Pa because this results in an inconsistency, with water vapor pressure values resulting from a calibration function based on this upper bound exceeding this boundary. We further disregard upper‐bound values above 2.5 Pa, because the highest values in *e* resulting from the application of our recalibration functions remains far below this value. Finally, we obtain the standard deviation of recalibrated water vapor pressure values using a range of calibration functions based on upper bounds between 1.5 and 2.5 Pa. This standard deviation never exceeds 15% of the water vapor pressure values obtained using the calibration function selected, equation (2). Figure 6 shows the independently added instrument errors and errors from the upper bound assumption for measurements obtained by the TECP on sols 55 and 56, nearly covering a full diurnal cycle. Note that the measurements between 6 and 8 a.m. were obtained at 0.8 m height while the others were obtained 3 cm above the ground (Zent et al., 2010). The error increases with water vapor pressure from a minimum at 0.005 Pa at 3 a.m. to its maximum of 0.3 Pa at noon. The relative error increases similarly from a minimum of 17% at 3 a.m. and 11 p.m. to 26% at noon. The bimodal behavior of the water vapor pressure shown in Figure 6 is not unique to sol 55 (see Figure 4) and may be explained by the north‐facing lander workspace and shadowing from the lander and/or the TECP itself, resulting in a temporarily lowered ground temperature (Zent et al., 2010) and less sublimation of exposed water ice in the workspace, lowering the water vapor pressure measured by the TECP 3 cm above the ground.

## 4 Comparison with Previous TECP RH Calibrations and Independent Measurements

**Figure 7:**
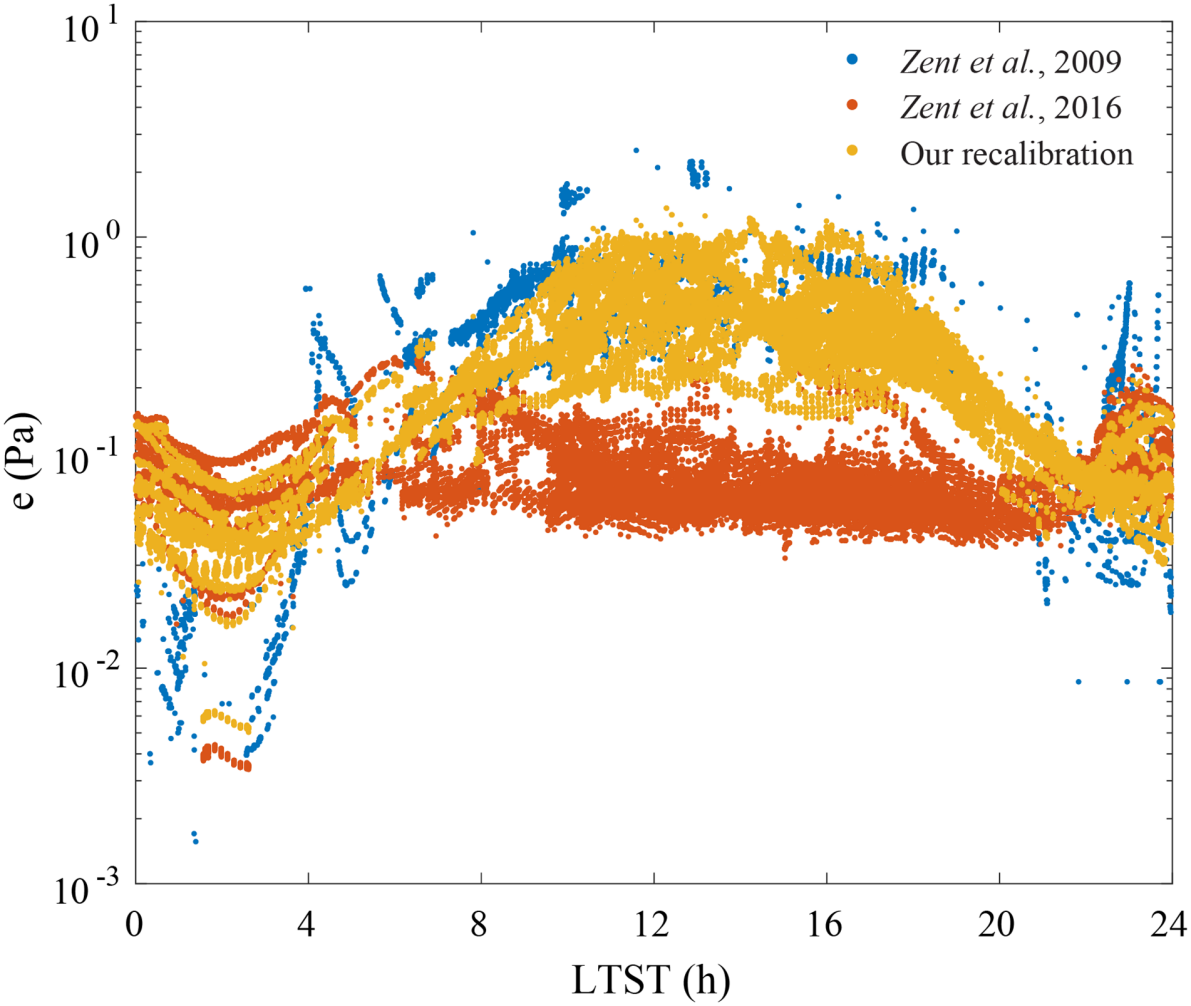
Comparison of our calibration of the thermal and electrical conductivity probe relative humidity sensor with previous calibrations.

Figure 7 compares the water vapor pressure values resulting from our TECP recalibration in yellow, the original preflight calibration in blue (Zent et al., 2010), and a postflight calibration in orange (Zent et al., 2016; current PDS values). While during nighttime, our calibration shows values that are in excellent agreement with those of the revised 2016 calibration, during daytime, our values are closer to those of the original calibration. This is because the revised calibration and that presented here have used the same set of in‐flight data to augment the original calibration at *T*_*b*_ < 208 K. On the contrary, while the revised calibration did not cover the warmest and driest conditions experienced during daytime, we exposed the TECP engineering unit to such conditions (Figure 2) using a range of different upper bounds for the frost point (225 to 213 K, corresponding to *e* values of ~5 to 1 Pa) and then performing sensitivity studies to check the robustness of the new calibration function in that range (section 3.3).

**Figure 8:**
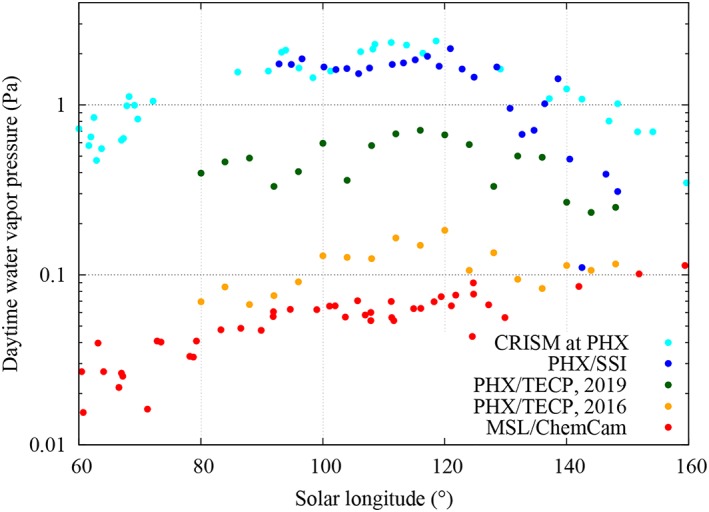
Comparison of the maximum diurnal water vapor pressure values throughout the Phoenix (PHX) mission obtained using the results of our recalibration (dark green), the previous postflight calibration (orange; Zent et al., 2016), and from precipitable water vapor column abundance retrievals at the PHX landing site by Compact Reconnaissance Imaging Spectrometer for Mars (CRISM; cyan) and PHX Surface Stereo Imager (SSI, blue; Tamppari et al., 2010). Also shown are water vapor pressure values derived around noon by the Mars Science Laboratory/Chemcam instrument (red; McConnochie et al., 2018). For the sake of clarity, PHX/thermal and electrical conductivity probe values (dark green and orange) shown in this figure correspond to averages over *ΔL*_*s*_ = 5° bins, and therefore absolute maximum values shown here are slightly lower than in Figure 4. CRISM and SSI measurements were taken at ~14:00 Local Mean Solar Time (LMST) and between 13:00 and 17:00 LMST, respectively.

To place our results in broader context, Figure 8 shows the maximum diurnal water vapor pressure values throughout the mission obtained using our recalibration (dark green), the 2016 postflight calibration (orange), and data from PWC retrievals at the PHX landing site made by the PHX's SSI (blue) and CRISM (cyan; Tamppari et al., 2010). In addition, to compare measurements by PHX with those by the MSL mission (4.5°S, 37.4°E), we also include water vapor pressure values derived from the ChemCam instrument (red) around noon (McConnochie et al., 2018) in Figure 8. While the intraseasonal variation is similar for each data set, our recalibrated values are in better agreement with those derived from SSI and CRISM. Moreover, water vapor pressure values obtained from our recalibration are significantly higher than those at the MSL site, as expected from other PWC retrievals for both landing sites during northern spring and summer (Tamppari et al., 2010; McConnochie et al., 2018).

## 5 Discussion

Direct measurements of the near‐surface RH on Mars have so far only been performed by the PHX/TECP and the Rover Environmental Monitoring Station (REMS) instrument onboard the MSL Curiosity rover (Harri et al., 2014).

While the MSL/REMS RH sensor has been operating successfully for more than 2,400 sols as of May 2019, providing complete coverage of the near‐surface RH from diurnal to interannual timescales (Harri et al., 2014; Martín‐Torres et al., 2015; Savijärvi et al., 2016; Martínez et al., 2016, 2017; Gough et al., 2018; Rivera‐Valentín et al., 2018; Savijärvi et al., 2019), daytime *e* values derived from these measurements are unreliable (Savijärvi et al., 2016). This is because of calibration uncertainties at the warmest conditions and the extremely low *RH* values measured at the MSL landing site during daytime (Martínez et al., 2016).

Since only the PHX/TECP can currently provide reliable daytime *e* values on Mars, and the main difference between the calibration presented here and that presented in Zent et al. (2016) is in daytime *e* values (Figure 6), the results shown here are important to shed light on the Mars near‐surface humidity environment, in particular, on the role played by the exchange of water vapor between the regolith and the atmosphere, and on the potential for brine formation at the PHX landing site. We discuss these two topics next.

### 5.1 Diurnal exchange of H_2_O between the regolith and the atmosphere

On a global scale, the exchange of H_2_O between the regolith and the atmosphere has been analyzed based on variations in PWC measured from orbit (Jakosky & Farmer, 1982; Smith, 2004; Fedorova et al., 2006; Fouchet et al., 2007; Melchiorri et al., 2007). While there seems to be consensus that the regolith seasonally exchanges water with the atmosphere (Jakosky, 1985; Houben et al., 1997; Böttger et al., 2005), assessments of the role of the regolith at diurnal timescales are more uncertain. Observed day‐to‐day variations in PWC in certain locations of Mars have been attributed to the exchange of water between the regolith and the atmosphere (Titov et al., 1994; Formisano et al., 2001). However, orbital measurements do not allow for a complete diurnal coverage nor resolve the atmospheric layers close to the ground where the exchange would occur. Moreover, some laboratory studies show that kinetics of H_2_O exchange between the regolith and atmosphere might be too slow to be significant at diurnal timescales (Zent et al., 2001).

On a local scale, the Imager for Mars Pathfinder was the first instrument to measure the atmospheric water on Mars from its surface, by taking images of the sun in the 0.94‐μm H_2_O band and deriving the atmospheric water column density. However, no significant diurnal variations were observed (Titov et al., 1999). Here, new results of water vapor pressure at the PHX landing site show strong evidence for significant exchange of H_2_O between the atmosphere and the regolith at diurnal timescales (Figure 4). First, the water vapor pressure undergoes a large diurnal variation of two orders of magnitude throughout most of the mission. For instance, the nearly complete diurnal coverage on sols 55 and 70 (Figure 3) indicates that the water vapor pressure values vary from around ~0.01 Pa (~10 ppmv) at 2–3 a.m. to ~1 Pa (~10^3^ ppmv) at noon. Second, water vapor pressure values decrease shortly after 16:00 (Figure 4) throughout most of the mission, well before the atmosphere or the regolith reach the frost point (Figure 5). Thus, since frost deposition and sublimation can be discarded, adsorption and/or salt hydration appear to be likely mechanisms exchanging H_2_O with the atmosphere at diurnal timescales. Unfortunately, independent, simultaneous TECP measurements of the soil wetness, necessary to prove the hypothesis of an active regolith, could not be achieved with enough certainty due to nonideal placement of the TECP needles in the soil (Zent et al., 2010). A more detailed analysis of TECP RH measurements (e.g., Rivera‐Valentin & Chevrier, 2015), maybe in combination with numerical modeling (e.g., Savijärvi & Määttänen, 2010), is needed to place further constraints on these mechanisms and will be the subject of future work.

**Figure 9:**
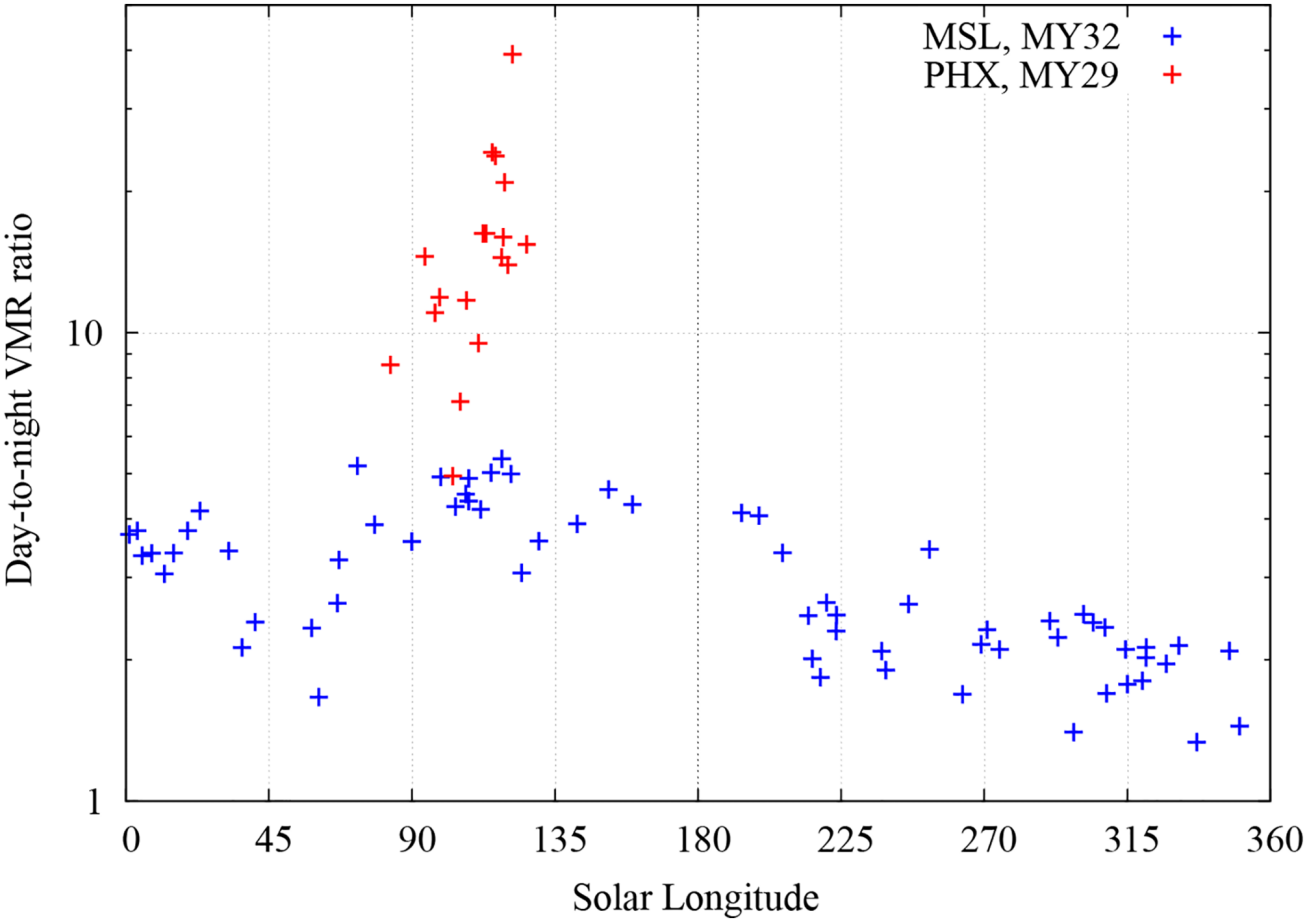
Day/night ratio comparison of water vapor pressure between the Phoenix (PHX, red) and the Mars Science Laboratory (MSL, blue) mission. At both landing sites and for every *L*_*s*_, the ratio is always >1, indicating higher daytime than nighttime values. At the PHX landing site, the ratios are one order of magnitude larger than at the MSL site, indicating larger atmosphere‐regolith H_2_O exchange.

To put diurnal variations of water content at the PHX site in a broader context, Figure 9 compares daytime to nighttime water vapor pressure ratios at the PHX and MSL landing sites. We place several requirements on the TECP water vapor pressure data used for this comparison, resulting in a small number of day/night ratios. Daytime values have to be obtained near the diurnal maximum, between 10:30 and 13:50 LTST, while nighttime values have to be near the minimum, between 00:00 and 04:00 LTST. Daytime and nighttime values are not available for each individual sol, so we use measurements at both times within five sols. Further, we only use values where the TECP height above the surface does not considerably change between day and night and is at least 0.4 m off the ground. MSL ratios were obtained from nighttime (~04:00–06:00) REMS and daytime (~noon) Chemcam measurements (Martínez et al., 2016; McConnochie et al., 2018). Figure 9 shows clearly larger day/night ratios of water vapor pressure at PHX compared to MSL, suggesting a stronger atmosphere regolith interchange. The day/night ratio seems to increase toward the end of the PHX mission with the approaching northern winter and colder nighttime ground temperatures, whereas the seasonal change of day/night water vapor pressure ratio is flatter at MSL, with a maximum at *L*_*s*_ ~ 100°, in the southern winter.

### 5.2 Brine formation potential

PHX TECP RH sensor data can shed light on the possibility of brine formation in the Martian polar region. Indeed, evidence for temporarily liquid brine was observed at the landing site in the form of droplets on the lander struts that changed location, size, and coloration, as well as soft ice in one of the dug trenches, suspected to be refrozen brine (Renno et al., 2009). Further, dielectric signatures in the subsurface (Stillman & Grimm, 2011) and the heterogeneous distribution of salts in the regolith (Cull et al., 2010) suggested the temporary existence of liquid brine.

Two mechanisms have been suggested for brine formation on Mars: the absorption of atmospheric water vapor by salts (deliquescence) when the RH exceeds a threshold value known as the deliquescence RH and the temperature is above the salts' eutectic value (Clark, 1978; Chevrier et al., 2009; Renno et al., 2009; Davila et al., 2010; Gough et al., 2011; Nuding et al., 2015; Nikolakakos & Whiteway, 2015, 2018), and ice melting when the temperature exceeds the eutectic value of salts in contact with water ice (Brass, 1980; Clark & Van Hart, 1981; Fairén et al., 2009; Marion et al., 2010; Fischer et al., 2014).

**Figure 10:**
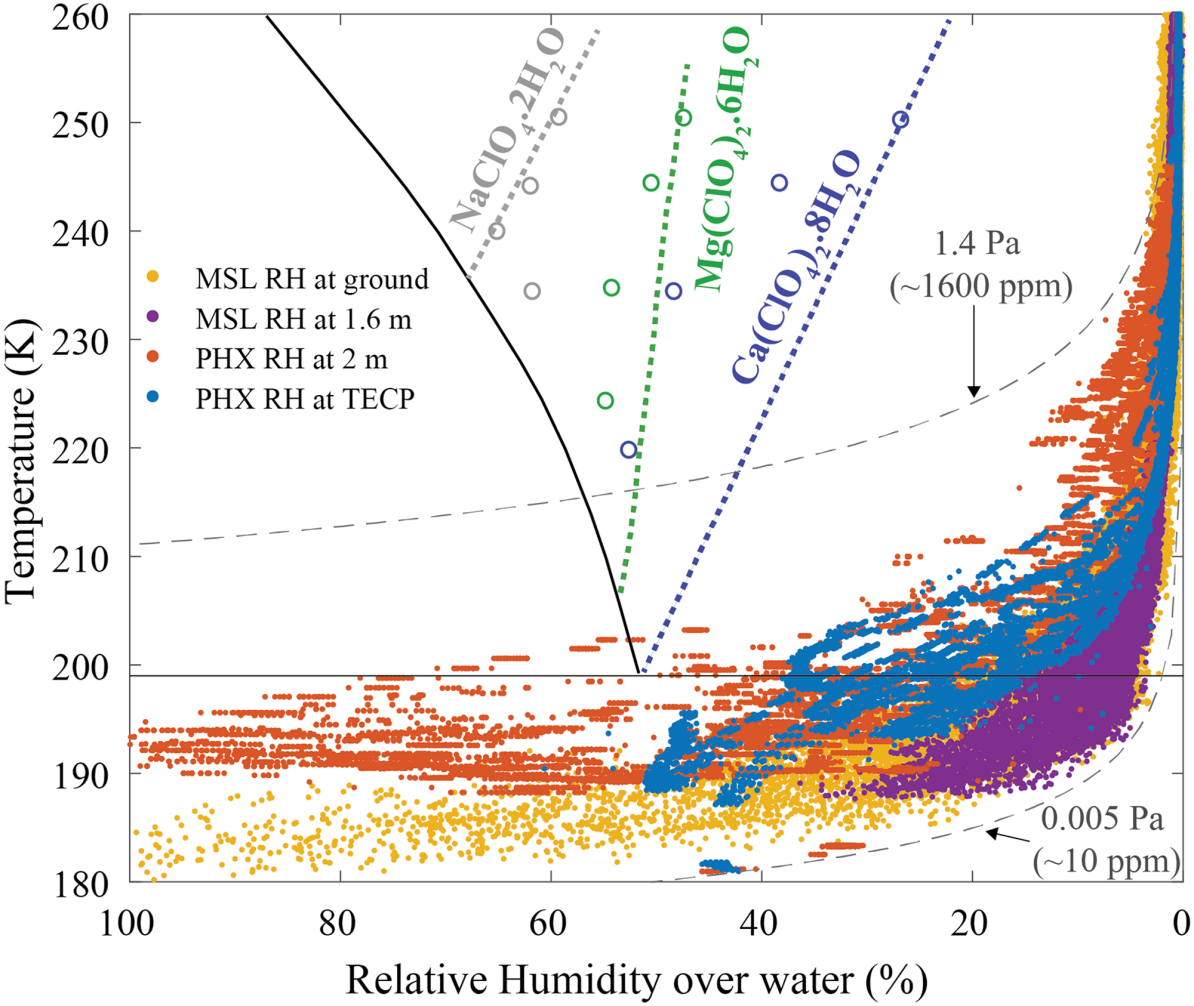
Stability diagram of NaClO_4_, Mg (ClO_4_)_2_, and Ca (ClO_4_)_2_ with superimposed values of Phoenix (PHX) relative humidity (RH) and temperature values at the thermal and electrical conductivity probe (TECP) location (blue) and at 2 m height (orange) and Mars Science Laboratory (MSL)/ Rover Environmental Monitoring Station values at the ground (yellow) and at 1.6 m height (purple). RH values shown here are converted to be with respect to liquid water for comparison with the brine stability lines, not with respect to water ice as measured by the instruments. For each salt, the colored thick‐dashed line represents the deliquescence relative humidity at which the various salts form aqueous solutions. Results from previous lab experiments of deliquescence of Ca, Mg, and Na perchlorates are shown in colored empty circles (Gough et al., 2011; Nuding et al., 2014). For reference, the eutectic temperature isotherm of Ca (ClO_4_)_2_ (solid black at ~199 K) and two isobars (dashed black) showing water vapor pressure values of 0.005 (minimum measured by the TECP) and 1.4 Pa (maximum measured by the TECP) are shown.

Figure 10 shows a stability diagram of sodium, magnesium, and calcium perchlorate salts present in the Martian regolith (Hecht et al., 2009; Kounaves et al., 2014), with superimposed values of temperature and RH over liquid water at the PHX landing site as well as for comparison at the MSL landing site. These perchlorates are relevant for brine formation on Mars because of their low eutectic temperatures and because they were found in polar and equatorial regions (Hecht et al., 2009; Glavin et al., 2013), suggesting that they are distributed globally. Brine is unlikely to form by deliquescence at the MSL site (yellow/purple) because of the low RH at temperatures above the salts' eutectic. Similarly, at the PHX site, the low RH at temperatures above the salts' eutectic at the TECP location (blue) makes deliquescence unlikely. At 2 m height (orange), where the air temperature measured by MET is the least influenced by artificial heating from the lander, the RH is high enough to cross the calcium perchlorate deliquescence line temporarily while the temperature is still above the eutectic. However, this only occurs during a short period of the day on a few sols, between 12 and 6 a.m., and it remains an open question whether kinetics of brine formation via deliquescence is rapid enough to occur during the short periods of the day when the conditions are favorable (Fischer et al., 2014). In fact, past experiments have shown that bulk brine formation by deliquescence at PHX surface conditions is less likely (Fischer et al., 2014), but that brine could readily form by contact of salts with the bulk ice present in the shallow subsurface (Fischer et al., 2016). Further studies show that subsurface conditions at the PHX and MSL landing sites may be conducive to temporary deliquescence (Primm et al., 2018; Rivera‐Valentín et al., 2018).

## 6 Conclusion

We have recalibrated the PHX/TECP RH sensor using data covering the entire range of temperature and RH conditions observed at the PHX landing site. Specifically, we have extended the postflight calibration obtained by Zent et al. (2016) to daytime conditions with very low RH and high temperature values.

The RH values resulting from our recalibration are in excellent agreement with independent observations by the RA camera and the LIDAR, showing nighttime frost formation from about sol 70 (*L*_*s*_ ~108°) and fall streaks and fog reaching all the way to the ground from sol 109 (*L*_*s*_ ~128°), respectively. Similarly, the highest maximum diurnal values of water vapor pressure obtained from our recalibration occur between sols 60 (*L*_*s*_ ~ 104°) and 90 (*L*_*s*_ ~ 118°), in excellent agreement with contemporaneous satellite retrievals of water vapor column abundance at the PHX landing site.

While during nighttime, our calibration shows values that are in excellent agreement to those of the revised 2016 calibration, during daytime, our values of water vapor pressure are one order of magnitude larger. We believe this is because while the revised calibration did not cover the warmest and driest conditions experienced during daytime, we exposed the TECP engineering unit to such conditions. Specifically, water vapor pressure values obtained from our recalibration are in the range ~0.005–1.4 Pa (~180–215 K frost point) while those obtained in Zent et al. (2016) are in the range ~0.004–0.4 Pa (~178–206 K) frost point.

Our daytime (upper bound) values are in better agreement with independent, contemporaneous estimations of water vapor pressure from the ground by the PHX/SSI instrument, and from orbit by CRISM, both of which show values of a few Pa. Also, our daytime values are significantly higher than those at the MSL site (which are as high as ~0.1 Pa), as expected in the northern polar region during northern spring and summer.

Since direct measurements of the near‐surface RH on Mars have only been performed by the PHX/TECP and MSL/REMS instruments, but daytime water vapor pressure values derived from MSL/REMS measurements are unreliable and need to be supplemented by MSL/Chemcam‐derived values, the results from our recalibration are important to shed light on the near‐surface humidity environment on Mars. Our results clearly show larger day‐to‐night ratios of water vapor pressure at PHX compared to MSL, suggesting a stronger atmosphere regolith interchange.

Our results show that the near‐surface environmental conditions for brine formation via deliquescence are barely achieved at the PHX landing site, where the necessary deliquescence temperature and RH are only exceeded for short times between midnight and 6 a.m. on a few sols. Possibly slow brine formation kinetics at low temperatures may inhibit any temporary brine formation. Nonetheless, conditions in the shallow subsurface may be more favorable for brine formation.

The results of this recalibration can lead to a better understanding of the hydrological cycle at the PHX landing site and the Martian northern polar region in general.
